# Professional nursing practice in critical units: assessment of work environment characteristics

**DOI:** 10.1590/1518-8345.1424.2854

**Published:** 2017-03-09

**Authors:** Luiz Felipe Sales Maurício, Meiry Fernanda Pinto Okuno, Cássia Regina Vancini Campanharo, Maria Carolina Barbosa Teixeira Lopes, Angélica Gonçalves Silva Belasco, Ruth Ester Assayag Batista

**Affiliations:** 1 Resident Nurse, Universidade Federal de São Paulo, São Paulo, SP, Brazil.; 2 PhD, RN, Escola Paulista de Enfermagem, Universidade Federal de São Paulo, São Paulo, SP, Brazil.; 3 MSc, RN, Escola Paulista de Enfermagem, Universidade Federal de São Paulo, São Paulo, SP, Brazil.; 4 PhD, Adjunct Professor, Escola Paulista de Enfermagem, Universidade Federal de São Paulo, São Paulo, SP, Brazil.

**Keywords:** Nursing, Professional Practice, Intensive Care Units, Emergency Medical Services, Health Facility Environment, Nursing Administration Research

## Abstract

**Objective:**

assess the autonomy, control over environment, and organizational support of nurses' work process and the relationships between physicians and nurses in critical care units.

**Method:**

cross-sectional study conducted with 162 nurses working in the intensive care units and emergency service of a university hospital. The workers' satisfaction with their work environment was assessed using Brazilian Nursing Work Index - Revised, translated and adapted for the Brazilian culture.

**Results:**

average age was 31.6 ± 3.9 years; 80.2% were women; 68.5% Caucasians and 71.6% worked in intensive care units. The nurses considered autonomy (2.38 ± 0.64) and their relationship with physicians (2.24 ± 0.62) to be characteristics of the work environment that favored professional practice. Control over environment (2.78 ± 0.62) and organizational support (2.51 ± 0.54), however, were considered to be unfavorable. No statistically significant differences were found between the units based on the scores obtained by the professionals on the Brazilian Nursing Work Index - Revised.

**Conclusion:**

autonomy, relationship between physicians and nurses, and organizational support were considered by the units to be characteristics that favored nurses' professional practices. On the other hand, control over environment and organizational support were considered unfavorable.

## Introduction

Critical care units, such as emergency rooms and intensive care units, are characterized by environments prepared to provide care to severely ill patients facing a potential risk of death, and are important components of healthcare provided in Brazil^1^.

The need for beds for critical patients has grown worldwide and is related to numerous factors, such as the growth of an aging population, increase in chronic diseases, and a change in the perception of what critical care might offer^2^ ^-^ ^3^.

One study conducted in the United States reports that the demand for and length of stay of severe patients in emergency services have increased in the last decade, consequently increasing the need for intensive care in these units, with the potential to lead to greater stress in places working above their capacity^4^.

In this complex context, the perceptions of these work environments may interfere in the functioning of these services and, for this reason, it is important to measure the characteristics that favor the professional practice of nurses so that interventions are implemented to support professional practice^5^.

The exercise of autonomy, control over work environment, and organizational support can differ throughout different health facilities. Additionally, professional relationships, the care delivery model, and management strongly influence the dynamics between healthcare workers and their work environment^5^ ^-^ ^8^. In this context, healthcare organizations constantly seek conditions to maintain and improve the quality of service provided, whether it is care delivery or administrative services^5^.

Therefore, it is important to consider a model for nursing professional practice characterized by a system composed of structure, processes, and values that support nurses in the delivery of care, as well as one that will aid in controlling the setting in which care is provided. This model is divided into subsystems, namely: a management system (decision-making structure and processes; a care delivery system (coordination of nursing work and assignment of tasks); professional values (values assigned to the code of professional ethics); professional relationships; remuneration and rewards (a system in which nurses are paid for their work and have their contributions, to the patients' and the organization's outcomes, acknowledged)^9^.

A working environment that is conducive to nursing practice contributes to higher levels of professional satisfaction and lower burnout levels. In regard to the patients, it decreases mortality rates and patients become more satisfied with care received, while the benefits for the institutions include decreased absenteeism and turnover of nursing professionals^10^.

The world literature reports evidence regarding the benefits of teamwork as an essential tool to maintain the quality of healthcare services. There are also positive associations concerning professional relationships, especially between nurses and physicians, nurses' autonomy, control over environment, and the organizational support provided to care delivery^7^ ^-^ ^8^.

The Nursing Work Index - Revised was translated and validated in Brazil seven years ago, but few studies were conducted to analyze the work settings of critical care units, which makes this study important to fill in this gap^5^ ^,^ ^11^.

Identifying the characteristics and the care delivery and management model that compose these services may improve care delivery by making it safer and more efficacious^8^. Given the previous discussion, this study's aim was to assess the autonomy, control over environment, and organizational support of nurses' work process and the relationships between physicians and nurses in critical care units.

## Method

This cross-sectional and analytical study was conducted in adult and pediatric emergency rooms and in 12 intensive care units: burn unit, medical clinic, cardiac surgery, cardiology, neonatal unit, pediatrics, pneumology, nephrology, general intensive care unit, neurosurgery, and the intensive care therapy unit of the emergency rooms, one private and one funded by the public system, of a university hospital located in São Paulo, Brazil. This study was approved by the Institutional Review Board at the Federal University of São Paulo (CAAE: 40730214.6.0000.5505) and the participants were included in the study after reading and signing free and informed forms.

The participants included nurses from the intensive care units and from the emergency rooms, while nurses from the Multidisciplinary Residency Program and those on sick leave or on vacation at the time of data collection were excluded, so that a total of 162 were included in the study.

Data were collected from December 2014 to May 2015 through a structured interview held in a private room, with a duration of 40 minutes on average.

The sociodemographic and professional variables were: sex, age, education, marital status, race, and family income, time since graduation, and time working in the institution. Professional satisfaction with work environment was assessed by the Brazilian Nursing Work Index - Revised, translated and adapted for the Brazilian culture^12^ ^-^ ^13^.

The Brazilian Nursing Work Index - Revised is composed of 57 items intended to measure the presence of certain characteristics in the work environment that favor nurses' professional practice. Fifteen items out of the total are conceptually distributed into three subscales: autonomy, control over environment, and relationship between nurses and physicians. Ten of these 15 items were grouped such that a fourth subscale was derived, the organizational support subscale^12^.

The subscale autonomy, which is composed of five items (4, 6, 17, 24 and 35) and the subscale control, composed of seven items (1, 11, 12, 13, 16, 46 and 48) represent the freedom nurses have to solve problems that affect the quality of nursing care.

The subscale relationship between nurses and physicians, composed of three items (2, 27 and 39), involves professional respect of establishing effective communication for the purpose of a common objective that concerns patient care.

The subscale organizational support is composed of ten items (1, 2, 6, 11, 12, 13, 17, 24, 27 and 48) derived from the aforementioned three subscales and is related to situations in which the organization supports nurses in the development of their professional practice.

The answers are classified on a four-point Likert scale: 1 - totally agree; 2 - partially agree, 3 - partially disagree, and 4 - totally disagree. The subscales' scores correspond to the average of the scores obtained by the respondents. Scores lower than 2.5 represent environments conducive to professional practice, while scores higher than 2.5 represent unfavorable environments; that is, the lower the score, the greater the presence of attributes that favor nurses' professional practice.

Descriptive analysis was used for sociodemographic and professional characterizations. Mean, standard deviation, median, minimum and maximum values were calculated for the continuous variables. Frequencies and percentages were computed for the categorical variables.

The association of continuous variables with the nurses' units was performed using Analysis of Variance. When necessary, the Chi-square test was used to associate the categorical variables and the Likelihood Ratio test.

Analysis of Variance was used to associate the subscales autonomy, control, relationship between physicians and nurses, and organizational support, while Spearman's coefficient of correlation was used for the continuous variables at a level of significance of 5% (p-value < 0.05).

## Results

The study included 162 nurses, aged 31.6±3.9 years old on average; 80.2% (n=130) were women; 68.5% (n=111) were Caucasian, and 61.1% (n=99) were single and 71.6% (n=116) worked in the intensive care unit.

Of the total of workers, 50.6% (n=82) had graduated more than five years ago; 86.4% (n=140) had attended a specialization program; 43.8% (n=71) had more than five years of professional experience, and 38.3% (n=62) had worked in the institution between one and three years.

In regard to income, 52.5% (n=85) had a personal income from three to five times the minimum wage, while the average number of individuals in a household depending on this income was 1.51 ± 1.39. Family income was reported by 71.6% (n=116) to be more than five times the minimum wage and the number of people depending on this income was 2.49 ± 1.55 per household.

Table 1 presents the comparison between the demographic variables of nurses working in the emergency room and those working in the intensive care units. This shows that most of those working in the emergency room were single, while those working in the intensive care units presented higher personal incomes, between three and five times the minimum wage, and also a larger number of dependents on both personal and family incomes.

**Table 1:** Comparison between the demographic variables of nurses working in the emergency room and intensive care units. São Paulo, SP, Brazil, 2014-2015 (n=162)

Variables		Unit		Total	p-value
		Emergency room n(%)	Intensive care unit n(%)	n(%)	
Marital status					
	Married	13(28.3)	33(28.4)	46(28.4)	0.0058*
	Single	33(71.7)	66(56.9)	99(61.1)	
	Stable union	-	8(6.9)	8(4.9)	
	Separated	-	9(7.8)	9(5.6)	
Personal income**^‡^**					
	From 1 to 3 times the minimum wage	9(19.6)	3(2.6)	12(7.4)	0.0003**^†^**
	From 3 to 5 times	17(37.0)	68W(58.6)	85(52.5)	
	More than 5 times	20(43.5)	45(38.8)	65(40.1)	
	Mean (standard deviation) of the number of dependents on personal income	1.1(0.9)	1.6(1.5)	1.5(1.3)	0.0407†
	Mean (standard deviation) of the number of dependents on family income	1.8(1.2)	2.7(1.6)	2.4(1.5)	0.0016†

*Chi-square test, †Analysis of Variance, ‡Minimum wage: R$ 788.00, Jan 1^st^ 2015, Brazil.

The scores obtained on the subscales of the Brazilian Nursing Work Index - Revised: autonomy, control over the environment, organizational support to the nurses' work process, and relationships between physicians and nurses in the critical care units are presented in Table 2.

**Table 2:** Mean and standard deviation of the scores obtained on the subscales of the Brazilian Nursing Work Index - Revised for nurses working in the emergency room and intensive care units. São Paulo, SP, Brazil, 2014-2015 (n=162)

Subscales Brazilian Nursing Work Index - Revised	Unit			Total
	Emergency room n(%)	Intensive care unit n(%)	n(%)
Autonomy	2.4(0.6)	2.3(0.6)	2.3(0.6)
Control over environment	2.8(0.6)	2.7(0.6)	2.7(0.6)
Relationships between physicians and nurses	2.2(0.5)	2.2(0.6)	2.2(0.6)
Organizational support	2.5(0.5)	2.4(0.5)	2.5(0.5)

No statistically significant differences were found between those working in the intensive care units and those in the emergency room when demographic and professional variables were associated with the subscales of the Brazilian Nursing Work Index - Revised.

## Discussion

The nurses addressed in this study presented an average age of 31.6 years old; 68.5% were Caucasian, and most were women (80.2%). These characteristics may be associated with nursing's historic employment trajectory^14^.

Most professionals worked in the intensive care unit (71.6%), had graduated more than five years ago (50.6%) and were specialists (86.4%). These findings corroborate the results of other studies^12^ ^,^ ^15^. The fact that most workers were specialists may be related to a requirement of critical units, which requires professionals to have greater qualification for providing care to patients with more complex conditions^7^ ^,^ ^12^ ^,^ ^16^.

The results concerning having more than five years of experience and having worked in the institution from one to three years are similar to those reported by other studies conducted in intensive care units^5^ ^,^ ^8^ ^,^ ^17^. These are relevant findings considering that inexperience is related to emotional burnout and malpractice, which leads to poor quality care provided to patients^18^.

Comparison between the sociodemographic variables of nurses working in the emergency room and intensive care units shows that most of those working in the emergency room were single. One study conduced in São Paulo, SP, Brazil in 19 intensive care units aiming to assess professional satisfaction among nurses reports that single workers more frequently sought professional development, implying that marital relations were established on average, at an older age^19^.

Additionally, most of those working in intensive care units presented a personal income between three and five times the minimum wage and a larger number of dependents on personal and family income. This finding shows that remuneration is one of the most important components to predict these professionals' levels of satisfaction with their work environment^20^.

When the characteristics of the work environment assessed by the Brazilian Nursing Work Index - Revised were analyzed in isolation, we verified that nurses from the intensive care units scored lower than 2.5 points for the subscales autonomy, relationship between physicians and nurses, and organizational support; that is, these professionals considered themselves to have autonomy, good relationships with physicians, and to be receiving organizational support. However they did not consider themselves to have good control over their environments, as they scored higher than 2.5 on this subscale.

The assessment of the 17 adult intensive care units of public, philanthropic and private facilities located in the interior of São Paulo based on the Brazilian Nursing Work Index - Revised resulted in a total average score of 2.13, a score lower than the one found in this study. Nonetheless, even though a higher score was obtained by the intensive care units assessed in this study, they still were characterized as having a work environment conducive to professional practice^7^.

No studies that applied the Brazilian Nursing Work Index - Revised to emergency services were found; in this study, assessment of this environment based on the Brazilian Nursing Work Index - Revised shows that only the subscales autonomy and relationship between physicians and nurses were scored lower than 2.5, while the total average score was 2.53, suggesting there is a need to implement interventions in these units to ensure greater professional satisfaction with the work environment and improved quality of care^7^.

The relationship between physicians and nurses was considered to be favorable in the units addressed in this study. Other studies conducted in intensive care units have also reported that a good relationship between physicians and nurses is one of the practices that most favor satisfaction with work environment when compared to autonomy, control over the environment, and organizational support. Additionally, this relationship may be associated with safer and improved care delivery^7^ ^,^ ^17^.

The subscale control over the environment, which assesses the freedom of nurses to solve problems that affect the quality of nursing care, both in intensive care units and emergency rooms, obtained the highest score compared to the remaining subscales, suggesting there is room for improvement regarding that issue. Studies conducted in intensive care units in the state of São Paulo, however, report that control over the environment was considered to be positive both in public and private institutions, indicating that the context is different from the one identified in this study^5^ ^,^ ^7^.

Comparison of the scores obtained by those working in the emergency room and intensive care units on the subscales of the Brazilian Nursing Work Index - Revised revealed no statistically significant difference. The scores obtained for the subscales regarding satisfaction with the work environment were analyzed in decreasing order, that is, from favorable to unfavorable, and the most favorable was the relationship between physicians and nurses, followed by autonomy, organizational support and control over environment. This finding corroborates those reported in the literature, showing control over the environment is a subscale that should be improved^7^.

One study was conducted in South Korea addressed 817 nurses from 39 intensive care units of 15 different hospitals in order to simultaneously identify the perceptions of nurses concerning the hospitals' and intensive care units' work environments. The subscales of the Nursing Work Index - Revised were applied and revealed discrepancies in the results of the hospitalization units in comparison to the intensive care units. That is, nurses working in the intensive care units were more satisfied with their work place than those working in the organization's general context^8^.

No statistically significant differences were found in this study when the sociodemographic and professional variables were associated with the subscales of the Brazilian Nursing Work Index - Revised. This lack of significance may be associated with the fact that both units provide care to critical patients and demand uninterrupted medical and nursing assistance, in addition to complex equipment and specialized human resources^21^ ^-^ ^23^.

Autonomy and relationship between physicians and nurses are the practices with the highest positive predictors favoring the professional practice of nurses in the work environment, whether they work in an emergency room or intensive care unit. Organizational support and control over the environment, however, need to be improved to enhance quality management by providing safer care to patients and by improving professional satisfaction with work environment^7^.

One study conducted in China verified that a work environment that values autonomy, a systematized care structure, management and leadership, is strongly linked to satisfaction at work. Hospitals that provide organizational support favor satisfaction on the part of workers and contribute to improved safety and quality of care delivery^24^.

Therefore, this study supports the development of a set of elements in the professional practice of nurses aiming to create improved working conditions by implementing quality management able to ensure efficient and safe care so as to attain an environment that attracts and retains professionals.

Therefore, this paper promotes reflection upon the professional practice of nurses in their work environment. Further studies are needed to acquire more knowledge on work environment, especially in emergency services, and how this interferes with professional practice.

This study's limitations involve the fact it was conducted in a single facility under public management and also the limited number of studies assessing the work environment in Brazilian contexts, which hindered comparisons. Despite these limitations, this study advanced knowledge concerning emergency rooms, the main entryway for patients into the public health system, as no studies pursuing the same objective were found. Emergency services face complex problems, such as high demand, high turnover, and stressed professionals. This study's results can contribute to better management of these units, by changing the institutional culture concerning factors that require improvement (control over the environment and organizational support), which in turn can improve the quality of care delivery.

## Conclusion

Both services presented favorable results for the subscales autonomy, relationship between physicians and nurses, and organizational support, all of which are characteristics of the work environment that favor nurses' professional practice. Control over environment and organizational support were reported as being unfavorable and requiring interventions to improve the critical units. This study's results present no differences between the work environments of the emergency room and intensive care units were compared based on the scores obtained in the Brazilian Nursing Work Index - Revised.

A situational diagnosis is an essential stage toward the implementation of interventions and establishment of a management model of nursing care practice with well-defined criteria.
